# *RB1 *gene mutation up-date, a meta-analysis based on 932 reported mutations available in a searchable database

**DOI:** 10.1186/1471-2156-6-53

**Published:** 2005-11-04

**Authors:** José R Valverde, Javier Alonso, Itziar Palacios, Ángel Pestaña

**Affiliations:** 1 Servicio de Informática. Centro Nacional de Biotecnología, CSIC. Campus de Cantoblanco. 28049-Madrid, Spain; 2 Oncolab. Deparatamento de Biología Molecular y Celular del Cáncer. Instituto de Investigaciones Biomédicas "A. Sols", CSIC-UAM. 28029 Madrid, Spain

## Abstract

**Background:**

Retinoblastoma, a prototype of hereditary cancer, is the most common intraocular tumour in children and potential cause of blindness from therapeutic eye ablation, second tumours in germ line carrier's survivors, and even death when left untreated. The molecular scanning of *RB1 *in search of germ line mutations lead to the publication of more than 900 mutations whose knowledge is important for genetic counselling and the characterization of phenotypic-genotypic relationships.

**Results:**

A searchable database (RBGMdb) has been constructed with 932 published *RB1 *mutations. The spectrum of these mutations has been analyzed with the following results: 1) the retinoblastoma protein is frequently inactivated by deletions and nonsense mutations while missense mutations are the main inactivating event in most genetic diseases. 2) Near 40% of *RB1 *gene mutations are recurrent and gather in sixteen hot points, including twelve nonsense, two missense and three splicing mutations. The remainder mutations are scattered along *RB1*, being most frequent in exons 9, 10, 14, 17, 18, 20, and 23. 3) The analysis of *RB1 *mutations by country of origin of the patients identifies two groups in which the incidence of nonsense and splicing mutations show differences extremely significant, and suggest the involvement of predisposing ethnic backgrounds. 4) A significant association between late age at diagnosis and splicing mutations in bilateral retinoblastoma patients suggests the occurrence of a delayed-onset genotype. 5) Most of the reported mutations in low-penetrance families fall in three groups: a) Mutations in regulatory sequences at the promoter resulting in low expression of a normal Rb; b) Missense and in-frame deletions affecting non-essential sequence motifs which result in a partial inactivation of Rb functions; c) Splicing mutations leading to the reduction of normal mRNA splicing or to alternative splicing involving either true oncogenic or defective (weak) alleles.

**Conclusion:**

The analysis of *RB1 *gene mutations logged in the RBGMdb has shown relevant phenotype-genotype relationships and provided working hypothesis to ascertain mechanisms linking certain mutations to ethnicity, delayed onset of the disease and low-penetrance. Gene profiling of tumors will help to clarify the genetic background linked to ethnicity and variable expressivity or delayed onset phenotypes.

## Background

Retinoblastoma (MIM# 180200), a rare embryonic neoplasm of retinal origin, is the most common intraocular tumor in children, with a relative incidence of 3% of all pediatric tumors. Although current therapeutic strategies have led to dramatic improvement of individual prognosis, retinoblastoma is still life-threatening when leaved untreated or in cases of late diagnosis, a condition of concern in developing countries [1]. The frequency estimates of retinoblastoma in different populations range between 1:34.000 and 1:10.000 live-born, with the most reliable figures between 1:28.000 and 1:15.000. An increasing incidence observed in recent studies can result from more complete ascertainment and also from population-genetic reasons, due to the increased survival of retinoblastoma patients [2]. Most of the clinical phenotypes can be explained by the double mutational inactivation of the retinoblastoma susceptibility gene [3], the prototype tumor suppressor gene that controls cell cycle progression [4]. However, additional mutations in apoptosis signaling may well be involved in tumor development [5], a hypothesis that has been in the cell-of-origin studies in mice [6]. In addition, a detailed analysis of the relations between genotype and phenotypic expression suggest that the hereditary retinoblastoma has features of a complex trait [7]. In the hereditary form of the disease, a germ line mutation is transmitted as a high penetrance (90%) autonomic dominant trait, resulting in a 45% risk in offspring of patients with hereditary retinoblastoma; the second inactivating mutation occurs in retinal cell precursors [8]. Most of these patients have bilateral retinoblastoma and a mean age at diagnosis of 12 months. In the non-hereditary form of the disease, both inactivating events occur during somatic development of retinal cells and result in the relatively late onset of a single tumor in one eye [9]. However, nearly 15% of the unilaterally affected patients have germ line *RB1 *mutations, representing a 45% risk for their offspring, and these patients cannot be clinically distinguished from patients with true somatic unilateral retinoblastoma, who present a negligible risk for siblings and offspring. Taking these situations together, the hereditary form represents nearly 50% of all the retinoblastoma patients, according to recent epidemiological figures [10]. The presence of *RB1 *germ line mutations confers an increased risk for development of second primary tumors in the survivors of hereditary retinoblastoma, with a cumulative incidence of 22% at the age of 25 years. Most of these second tumors were osteosarcomas (37.0%), other sarcomas (16.8%) and melanomas (7.4%), while brain tumors (4.5%), leukemia (2.4%) and non-Hodkin lymphomas (1.6%) were less frequent [11]. In addition, hereditary retinoblastoma survivors have a lifetime risk of developing common epithelial cancers [12].

The development of sensitive and reliable genetic tests to detect *RB1 *mutations has greatly improved the identification of carriers, facilitating accurate genetic counseling. In addition, the detection of children at risk among siblings would obviate the need for many routine examinations and potentially decrease the economic impact of the disease [13]. Attempts by several groups to define the mutations resulting in the inactivation of the *RB1 *gene in retinoblastoma have led to the identification of a broad spectrum of mutations. To date, the most comprehensive report of *RB1 *mutations corresponds to Lohman's database [14] which contains 228 different mutations and 130 recurrences. In this article we describe an up-dated, searchable database containing 500 distinct somatic or germ line *RB1 *mutations and more than 400 recurrences that we have retrieved from publications. In addition, we analyze the spectrum of *RB1 *mutations, with emphasis in molecular epidemiology and phenotype-genotype relationships. This information is important for the development of rapid procedures to detect mutations in patients and also to understanding the molecular mechanisms leading to tumors with different degrees of penetrance or expressivity.

## Results and discussion

The scope of the database of *RB1 *gene mutations (RBGMdb) is to retrieve and arrange data from the literature in a flexible and standardized electronic format as described in methods. In its present version, it contains 932 entries extracted from 68 articles referred in Additional File 1, together with the number of mutations they contribute and the country of origin of the reporting group. Out of these entries, 753 correspond to germ line mutations, 155 are somatic mutations in retinoblastoma patients and 24 correspond to *RB1 *somatic mutations found in other tumors. The distribution of these mutations in different retinoblastoma patient is shown in Table 1. These figures cannot be considered representative of the true incidence of retinoblastoma phenotypes, since most germ line studies were carried out in bilateral retinoblastoma patients and mutation analysis were performed in a limited amount of unilateral tumor samples. The database also gives information about the sex of the patient (140 entries) and the age at diagnosis or treatment (258 entries).

**Table 1:** Distribution of germ line and somatic *RB1 *mutations by phenotype of the patients.

	Germ line	Somatic	Total
Bilateral sporadic	424	24	448
Bilateral familiar	99		99
Low penetrance	27		27
Unilateral sporadic	52	131	183
Unilateral familiar	6		6
Not reported	145		145
Total	753	155	908

### Type of mutation

The RBGMdb contains 500 distinct mutations and 433 recurrences (see Table 2). Most of the 932 entries (42 %) correspond to nonsense (NS) point mutations. This figure is reduced to 18% of the sample when the 302 recurrent NS are omitted. In this case, the proportion of NS mutations is closer, although significantly different, from the data logged in the Human Gene Mutation Database [15], which gives information of more than 46000 mutations in human disease. In contrast to the high recurrence of NS mutations in retinoblastoma (70% of the total recurrences), small insertions, deletions or complex ins_del (ins/del) show a low recurrence and represent a high proportion (48%) of the 500 distinct *RB1 *mutations; this figure is much higher than the data in HGMD. Splicing mutations in *RB1 *have a moderate (19%) recurrence and represent twice as much of the proportion of the distinct SP mutations found in HGMD. On the contrary, MS *RB1 *mutations are very low (10% of the total) as compared with the 50% given in HGMD.

**Table 2:** Number of entries by mutation type as compared with HGMD data. Statistical analysis of distinct RBGMdbDB vs. HGMD using χ^2 ^test.

	All mutations		Distinct mutations		Distinct mutat.		
	RBGMdb	%	RBGMdb	%	HGMD	%	χ^2 ^test

Nonsense	395	42.4	93	18.6	5840	12.6	P < 0.0001
Missense	81	8.7	50	10.0	22940	49.6	P < 0.0001
Splicing	194	20.8	111	22.2	4771	10.3	P < 0.0001
Regulatory	7	0.7	5	1.0	626	1.4	P = 0.6267
Small ins/del	255	27.3	241	48.2	12076	26.1	P < 0.0001
Total	932		500		46253		

### Hot spots of *RB1 *gene mutations

**Figure 1:**
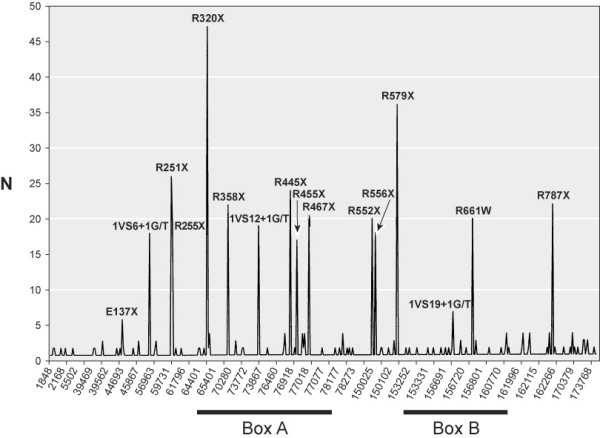
Mutational hot spots in RB1. The number of entries found for each mutation is represented against the modified genomic nucleotide. Description of high recurrent mutations is shown.

As shown in Figure 1, *RB1 *mutations are scattered along the genomic sequence, but also accumulate in discrete spots of high recurrence, comprising twelve nonsense mutations, three affecting splicing sites and two missense. These results confirm and extend previous observations [13,14] with new hot spots and higher recurrence figures. Most of the recurrences (270 out of 341, 79%) correspond to C to T transitions in eleven CGA-arginine codons, in exons 8, 10, 11, 14, 15, 17, 18 and 23 (see Additional File 2), but no mutations were found in three other CGA codons, located in exons 1 and 27. It is generally admitted that the hyper mutability of CGA codons depends on the methylated status of CpG dinucleotides and the spontaneous deamination of 5 methyl cytosine to thymidine [16]. In four of the mutated CGA codons (R251 and R255 in exon 8, R451 and R455 in exon 14) a high frequency of constitutive methylation (see Additional File 2) has been demonstrated and it is assumed that constitutive hyper methylation would also be present in the other CGA hot spots [17]. The absence of mutations at the CGA codon (R7) in exon 1, whose predicted consequence would be a short and inactive peptide, fits in this model since this codon is part of an unmethylated CpG island in the *RB1 *promoter [18]. On the other hand, the absence of mutations in two highly methylated CGA codons (R908 and R910) in exon 27 is expected, in view of the fact that mutations in the last *RB1 *exon are not oncogenic [18]. The same argument can apply to the absence of C>T mutations in the highly methylated CpG codons in position 451 (CGC), 855 (AGC), 857 (CGT) and 876 (CGC) in *RB1 *gene [17], whose predicted outcome would be non oncogenic missense or silent amino acid substitutions (R451R, S855S, R857C and R876R).

In this sense it is worth to mention the different fate of three CGG-codons in *RB1 *gene: two of unknown methylated status in exon 20, comprising the highly recurrent R661W (20 mutations as shown in Additional File 2) and R656W (1 mutation); the third, in exon 8 (R262) is frequently methylated although no mutations have been so far observed. In these cases, the differences in mutability cannot be explained by the methylation status not by differences in tumorogenity (oncogenity), since both R656W and R661W lie in the folded and hydrogen-bond rich structural domain of the A-B interface, together with other missense mutations [19]. Alternatively, the hyper mutability found in R661 can be explained by neighboring-nucleotide effects (**T**CGG in R661 versus **A**CGG in R656 and R 262) and the known differences in mutability of these tetranucleotides [20]. In the case of other non-CpG hot spots in *RB1 *gene, such as the nonsense E137X and the three splicing mutations affecting the first invariant nucleotide in introns 6, 12 and 19, the presence of short quasi-repeat sequences could be documented (see Additional file 2). These sequence motifs would favor replication errors such as misinsertions or misalignments leading to base substitution or single-base frameshift with the mismatch repair machinery [21]. Although this DNA environment offers an attractive explanation for the presence of hot spots in non CpG sites, no preponderance of direct or inverted repeats has been observed in the spectrum of single-base-pair substitutions logged in the HGMD [22]. However, the HGDM only includes distinct mutations and therefore an association of repeated sequence motifs with recurrent mutations cannot be excluded.

### Mutational spectrum of *RB1 *by exon

**Figure 2:**
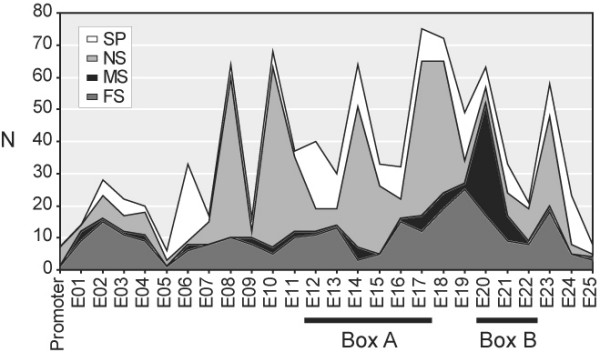
Spectrum of RB1 mutations by exon. The number of entries for each type of mutation is distributed by exon and adjacent 5' and 3' intronic sequences.

In addition to hot spots, frameshift and point mutations leading to amino acid substitutions or splicing are scattered along the retinoblastoma cDNA and non-coding adjacent splicing sites, giving the spectrum of mutations shown in Figure 2. With the exception of exons 5, 14, 15, 24, 25 and the non-mutated exons 26 and 27, frameshift mutations are randomly distributed through the *RB1 *coding sequence. Splicing mutations are also evenly distributed, but show preference for intronic sequences adjacent to exons 6, 12, 16, 17, 19 and 24; three of them are associated to the above described recurrences (see Figure 1). The exonic distribution of point mutations correspond to the hot spots already described in Figure 1. It is worth to mention that most missense substitutions (60 %) are located in cyclin box B, underlined by exons 19 to 21. The spectrum of *RB1 *mutations by exon has important implications in the mutational screening of retinoblastoma patients, which might benefit from the sequential analysis employing quantitative multiplex PCR (QM-PCR) methodology to detect frameshift mutations[23,24] and PCR-sequencing of highly mutated exons 9, 10, 14, 17, 18, 20 and 23.

### Spectrum of *RB1 *gene mutations by country of origin

**Figure 3:**
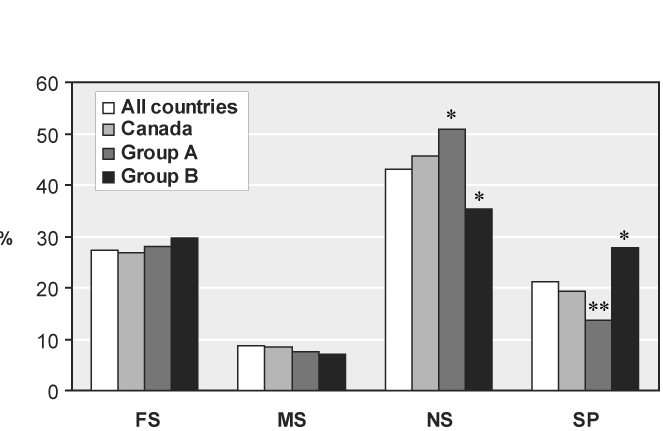
Spectrum of RB1 mutations by country of origin. Distribution of mutations in Canada and two groups of nations and statistical comparison with all mutations in RBGMdb. (*) and (**) stand for P < 0.05 and 0.01, respectively).

The distribution of mutations logged in the RBDB by type and country of origin (shown in Additional File 3) allows establishing the two different spectra of *RB1 *gene mutations shown in Figure 3. In certain South American countries (Argentina, Brazil, Colombia, Cuba and Ecuador) as well as in Russia, United Kingdom and Germany, amounting to a total of 392 mutations (group A in Figure 3), the incidence of NS and SP mutations is respectively higher (p = 0.017) and lower (P = 0.003) than in the grand total of 925 mutations. On the contrary, in United States, France and Spain (group B in Figure 3) the incidence of NS is lower (P = 0.022) and that of SP is higher (P = 0.023) than the average found for all mutations. The differences in incidence of NS (50.8 and 35.4%, P = 0.0002) and SP (13.7 and 27.9%, P < 0.0001) mutations between groups A and B respectively, are extremely significant and suggest the presence of predisposing ethnic backgrounds. Since most NS mutations in *RB1 *(80% in RBGMdb) correspond to C>T transitions, origin of a G: T mispair, and the eukaryotic mismatch (MMR) complex MSH2–MSH6 (MIM#120435 and 600678, respectively) seems to be more efficient in G: T mismatch repair [25], it is suggested that susceptibility to NS *RB1 *mutations can be increased by an imbalance between DNA methylation vs. mismatch repair (MSH2–MSH6) activities [26].

### Age and type of mutations

In correspondence with larger statistical studies [2,9] the mean age at diagnosis for bilateral and unilateral retinoblastoma patients in DBRB is of 12.5 and 24.8 months respectively, and this difference was extremely significant (P = 0.00006). Similar differences are observed in all but splicing mutations (Table 3), whose mean age at diagnostic in bilateral and unilateral patients are statistically indistinguishable. This result, which confirms previous observations [27], suggests that splicing mutations can be associated to a delayed onset phenotype. The molecular basis of this phenotype could be related to mechanisms considered in low-penetrance splicing mutations.

**Table 3:** Age at diagnosis in retinoblastoma patients according to phenotype and type of mutation. Statistical analysis using the Welch's unpaired t test.

Phenotype	Frameshift	Nonsense	Missense	Splicing	SP vs. NS-MS-FS
Unilateral	21.1	26.0	23.7	26.7	P = 0.7237
Bilateral	9.5	11.8	9.4	19.3	P = 0.0025
All	12.4	15.6	16.0	21.8	P = 0.0044
Unilateral vs. Bilateral	P = 0.0073	P = 0.0147		P = 0.3321	

### Low penetrance retinoblastoma

In 27 out of the 133 (20%) familial retinoblastoma entries in RBGMdb, the presence of unaffected carriers (reduced penetrance) or unilateral retinoblastoma or benign retinoma (reduced expressivity) were documented (see Additional file 4 for a complete description of the mutations and references). This figure probably represents an overestimation of the true incidence of the low penetrance (LP) phenotype, biased by its notorious scientific interest. As shown in Table 3, most of the reported mutations associated to LP families (23/28 = 82%) correspond to MS and SP mutations, with hot spots in g.156713 C>T (R661W), g.160757T>C (C712R) and g.45867G>T (IVS6+1G>T). Different mechanisms have been proposed to explain this rare phenotype, including epigenetic events, delayed mutation, involvement of a second retinoblastoma locus ("three-hit hypothesis") or host resistant factors. However, most low-penetrance retinoblastoma can be explained by mutations at the *RB1 *locus [28]. Mutations affecting regulatory sequences in the *RB1 *promoter are known to reduce the expression of normal Rb protein below a threshold level, necessary for tumor suppression functions [29,30]. Missense single-nucleotide substitutions can, under certain circumstances, partially inactivate the retinoblastoma function. In the case of the highly recurrent R661W mutant allele, Otterson et al. have shown that the mutant retinoblastoma protein has a temperature-sensitive pocket activity whose reversible fluctuations may result in the low penetrance phenotype. Temperature-sensitive Rb pocket activity may also explain the low penetrance of C712R and delN480 [31]. In the case of the large in frame deletions outside the pocket domain, such as Del: E04 [32] and Del: E24–25 [33], unessential functions of Rb protein seems to be affected.

After the report of a splicing mutation (c.2211G>A) affecting the last nucleotide of exon 21 [34], a new category of low-penetrance genotype has been proposed to occur through alternative splicing mechanisms. In the case reported by these authors, the RNA analysis showed skipping of exon 21 and a low amount (10%) of normally spliced RNA (E732E) which can explain the variable expressivity observed in that family. A similar mechanism can explain the low-penetrance of other splicing mutations affecting the last exonic nucleotide, such as c.1331A>G and c.1960G>C. In these cases, the splicing machinery could alternate between defective missense splicing (Q443P and V654L, respectively) and inactivating skipping of exons 13 or 21, both in the pocket box domain of Rb. In the low-penetrance family studied by Lefevre et al., a T>C substitution in the polypyrimidine tract of intron 8 (IVS9-10T>C) was shown to be at the origin of the in frame deletion of exon 9 giving a defective or inactive Rb protein lacking 26 amino acids from the N-terminal region. It is also possible that this mutation only partially affected spliceosome assembly and that the mutated allele could be in part correctly spliced. While this alternative splicing mechanism would better explain the low-penetrance phenotype, no supporting evidence is available [35]. Similar alternative mechanism could also explain the low-penetrance of c.2325+5G>A, causing in frame skipping of exon 22 [36]. In this case, the G>C transversion makes a slight reduction of the consensus value of the 5' splice site [37] from 88 to 75 compatible with the presence of a fraction of correctly spliced mutant allele.

Alternative splicing mechanisms might also be involved in the recurrent c.607+1G>T transversion, firstly described in a low-expressivity and delayed onset phenotype in one Spanish family [27], and thoroughly analyzed by Klutz et al [38] in two low-penetrance German pedigrees. These authors made the interesting observation of a posttranscriptional mechanism that reduces the level of the mutant transcripts (skipping of exon 6 giving a stop codon in exon 7) when the mutant allele was received from the father. However this parent-of-origin effect does not explain the low-penetrance phenotype, since all but one unaffected carriers have maternally inherited mutant alleles and high level of expression, while most of the tumor bearing carriers have paternally inherited mutant alleles and low level of expression of the mutant allele. In a search for alternative splicing mechanisms, using the electronic exon-search facilities at HGMP, we have observed the presence of a cryptic exon whose usage in mutant alleles (see Additional File 5) could result in a defective Rb protein lacking 12 amino acids in the amino terminal region. Since no evidence of in-frame restoring mechanisms in lymphoblastoid cell line derived from unaffected carriers was observed [38], the hypothetical alternative splicing mechanism should be explored in retinoblastoma derived cell lines. In the only nonsense substitution (Q675X) observed in a low-penetrance phenotype, the G>T transversion in c.2023 could also activate a cryptic splicing site involving the stop codon, with the result of a defective Rb lacking 22 amino acids [39].

## Conclusion

The analysis of *RB1 *gene mutations logged in the RBGMdb has shown relevant phenotype-genotype relationships and provided working hypothesis to ascertain mechanisms linking certain mutations to ethnicity, delayed onset of the disease and low-penetrance. In considering the variable phenotypes associated to low-penetrance genotypes (see frequencies in Table 4) Richter et al. have proposed that unilateral sporadic carriers of these mutations should be considered the founders of low-penetrance families [23]. The same observation has led to Lohmann et Galli to suggest that the hereditary retinoblastoma has features of a complex trait [7]. In order to clarify these alternatives, functional studies should be carried out in order to provide better insights into the proposed mechanisms for low-penetrance mutations. In addition, gene expression profiling of tumors will help to clarify the genetic background linked to ethnicity and variable expressivity or delayed onset phenotypes. It will also be desirable to build up an international retinoblastoma study group in order to gather high quality information relevant to molecular studies, prognosis, therapy response and long-range follow-up of carriers of low-penetrance mutations.

**Table 4:** Molecular basis of low penetrance retinoblastoma

Type of mutation	Number of LP families	Description of mutations and frequency ^a^	Functional consequences
Regulatory	3	-198G>A (1/2) -188G>T (1/2) -149G>C (1/1)	Low expression of normal Rb protein
MS point mutations	12	R661W (8/20) C712R (2/5) W563L (1/1) R787Q (1/1)	Partial inactivation of Rb protein
Inframe deletion	3	Del:N480 (1/1) Del:E04 Del: E24–25	Partial inactivation of Rb protein
Splicing	10	607+1G>T (4/11) 862-10T>C (1/1) 539+1delG, del E05 (1/1) 2325+5G>A, del E:22 (1/1) Q443P/del:E13 (1/1) V654L/splice (1/2) E732E/del E:21(1/2)	Alternative splicing and/or unessential exon skipping resulting in low expression or partial inactivation of Rb protein
NS point mutation	1	Q675X (1/1)	Alternative splicing involving the stop codon

^a^Ratio of mutations found in LP families vs. all mutations in the database is shown in brackets

## Materials and methods

### Data mining

Primary bibliographic resources were retrieved from Entrez-PubMed, searching for human retinoblastoma (*RB1*) mutations. Reprints of all these articles were obtained and additional articles describing *RB1 *mutations were picked up from its reference lists. In addition, all the articles from the same authors or research group were thoroughly scrutinized in order to avoid repetitions of mutations present in one patient. In all, data from 68 research articles have been compiled in the data base. These articles, together with the number of mutation they contributed and the country of origin of the main research group are supplied in the Additional file 1.

### Description of the flat-file format

All the mutations were thoroughly revised according the recommended nomenclature for sequence variations [40] using the genomic sequence GenBank: L11910.1, the cDNA sequence NCBI: NM_000321.1, and the protein sequence NCBI: NP_000312.1. The mutations were annotated in a Microsoft Excel data sheet containing 15 columns (Shown in Additional File 6).

### Structure and management of the database

To facilitate access to the collected data, we have created an SQL database and developed an easy to use web interface that provides for simple and complex queries to the database, allowing to sort results by any field and to produce both, HTML and PDF reports. The database engine is based on MySQL [41], a popular, open source DBMS. The user interface is based on HTML forms and PHP [42] scripts, with some bits of public Javascript code [43] for data validation and online help. PDF output is generated by using the FPDF library [44]. The whole code of the web-based user interface and SQL schema is publicly available from the site.

The public interface of RBGMdb is located at EMBnet Spanish node [45]. As we have already mentioned, it provides resources for issuing simple queries for records containing a search term, as well as for complex queries where up to four search terms may be looked up on user-specified fields each combined with its logical operator. Searches produce their results as an HTML table with online help that is displayed whenever the cursor moves over any field. Results may be further sorted by any field by just clicking on the field name in the table header. The user is offered the possibility of generating PDF reports for download and/or printing at every step of the process. In addition to the search forms, we have created a submission form to facilitate the public addition of new data. This form sends an e-mail to the database coordinator who effectively acts as supervisor for all new additions. Restricted access database update forms are also available for the database coordinator(s) to actually modify the data in the database.

### Computer analysis and statistics

When indicated, normal and mutated *RB1 *sequences were analyzed with the programs for exon identification available at the Computing Services of UK HUMAN GENOME MAPPING PROJECT [46]. Statistical significance tests used in this study included Chi square and Fisher exact test for 2 × 2 tables, and the Welch's unpaired t test and nonparametric Mann-Whitney test for the analysis of means. Statistical summaries from the Human Gene Mutation Database (HGMD) were retrieved from the web.

## List of abbreviations used

RBGMdb (Retinoblastoma gene mutation database), LP (low-penetrance), NS (nonsense), MS (missense), FS (frameshift), SP (splicing),

## Authors' contributions

JRV designed and constructed the database; JA and IP helped in the acquisition of data and the revision of the description of mutations; AP designed and coordinated the study, and drafted the article

## Supplementary Material

Additional File 1 List of contributing authors to RBGMdb with indication of country of origin of the main research group, number of mutations and reference. Click here for file

Additional File 2 Table of recurrent mutations with indication of sequence motifs and methylation status. Click here for file

Additional File 3 Table with the distribution of mutations by country of origin of the patients. Unless otherwise stated in the original publication, it is assumed that the country of origin of the patients correspond to the country of the main authoring group. Click here for file

Additional File 4 Table listing mutations found in low-penetrance families Click here for file

Additional File 5 Possible alternative splicing in the low-penetrance mutation c.607+1G>T Click here for file

Additional File 6 Description of the flat-file format of RBGMdb. Click here for file

## References

[B1] Zucker JM, Desjardins L, Doz F (1998). Retinoblastoma. Eur J Cancer.

[B2] Vogt F (1979). Genetics of retinoblastoma. Hum Genet.

[B3] Cavenee WK, Dryja TP, Phillips RA, Benedict WF, Godbout R, Gallie BL, Murphree AL, Strong LC, White RL (1983). Expression of recessive alleles by chromosomal mechanism in retinoblastoma. Nature.

[B4] Kaelin WG (1999). Functions of the retinoblastoma protein. Bioessays.

[B5] Gallie BL, Campbell CH, Devlin H, Duckett A, Squire JA (1999). Developmental basis of Retinal-specific induction of cancer by RB mutation. Cancer Res.

[B6] Chen D, Livne-bar T, Vanderluit JL, Slack RS, Agochiya M, Bremner R (2004). Cell-specific effects of Rb or RB/p107 loss on retinal development implicate an intrinsically death-resistant cell-of-origin in retinoblastoma. Cancer Cell.

[B7] Lohmann DR, Gallie BL (2004). Retinoblastoma: revisiting the model prototype of inherited cancer. Am J Med Gene Part C.

[B8] Knudson AG (1971). Mutation and cancer. Statistical study of retinoblastoma. Proc Natl Acad Sci USA.

[B9] Draper GJ, Sanders PA, Brownbill, Hawkins MM (1992). Patterns of risk of hereditary retinoblastoma and applications to genetic counseling. Br J Cancer.

[B10] Murphree AL (1995). Molecular genetics of retinoblastoma. Ophtalmol Clin North America.

[B11] Moll AC, Imhof SM, Schouten-Van Meeteren AYN, Kuik DJ, Hofman P, Boers M (2001). Second primary tumors in hereditary retinoblastoma: A register-based study, 1945–1997. Ophthalmology.

[B12] Fletcher O, Easton D, Anderson K, Gilham C, Jay M, Peto J (2004). Lifetime risks of common cancers among retinoblastoma survivors. J Natl Cancer Inst.

[B13] Harbour JW (1998). Overview of RB gene mutations in patients with retinoblastoma. Ophthalmology.

[B14] Lohmann DR (1999). *RB1 *gene mutations in retinoblastoma. Hum Mutat.

[B15] Stenson PD, Ball EV, Mort M, Phillips AD, Shield JA, Thomas NS, Abeysinghe S, Krawczak M, Cooper DN (2003). Human Gene Mutation Database (HGMD): 2003 update. Human Mut.

[B16] Cooper DN, Krawczak M (1989). Cytosine methylation and the fate of CpG dinucleotides in vertebrate genomes. Hum Genet.

[B17] Mancini D, Singh S, Ainsworth P, Rodenhiser D (1997). Constitutively methylated CpG nucleotides as mutation hot spots in the retinoblastoma gene. Am J Hum Genet.

[B18] Lohmann DR, Brandt B, Hopping W, Passarge E, Horsthemke B (1996). The spectrum of *RB1 *germ-line mutations in hereditary retinoblastoma. Am J Hum Genet.

[B19] Lee J-O, Russo AA, Pavletich NP (1998). Structure of the retinoblastoma tumour-suppressor pocket domain bound to a peptide from HPV E7. Nature.

[B20] Ollila J, Lappalainen I, Vihinen M (1996). Sequence specificity in CpG mutation hotspots. FEBS Lett.

[B21] Maki H (2002). Origins of spontaneous mutations: Specificity and directionality of base-substitutions, frameshift, and sequence-substitution mutagenesis. Annu Rev Genet.

[B22] Krawczak M, Ball EV, Cooper DN (1998). Neighboring-nucleotide effects on the rates of germ-line single-base-pair substitutions in human genes. Am J Hum Genet.

[B23] Richter S, Vandezande K, Chen N, Zhang K, Sutherland J, Anderson J, Han L, Panton R, Branco P, Gallie B (2003). Sensitive and efficient detection of *RB1 *gene mutations enhances care for families with retinoblastoma. Am J Hum Genet.

[B24] Houdayer C, Gauthier-Villars M, Lauge A, Pages-Berhouet S, Dehainault C, Caux-Moncoutier V, Karczynski P, Tosi M, Doz F, Desjardins L, Couturier J, Stoppa-Lyonnet D (2004). Comprehensive screening for constitutional *RB1 *mutations by DHPLC and QMPSF. Hum Mutat.

[B25] Kolodner RD, Marsischky GT (1999). Eukaryotic DNA mismatch repair. Curr Opin Dev Biol.

[B26] Lengauer C, Kinzler KW, Vogelstein B (1998). Genetic instabilities in human cancers. Nature.

[B27] Alonso J, Mendiola M, García-Miguel P, Abelairas J, Sarret E, Vendrell T, Navajas A, Pestaña A (2001). Spectrum of germline *RB1 *gene mutations in Spanish retinoblastoma patients. Phenotypic and molecular epidemiological implications. Hum Mutat.

[B28] Harbour JW (2001). Molecular basis of low-penetrance retinoblastoma. Arch Ophthalmol.

[B29] Sakai T, Ohtani N, McGee TL, Robbins PD, Dryja TP (1991). Oncogenic germ-line mutations in Sp1 and ATF sites in the human retinoblastoma gene. Nature.

[B30] Cowell JK, Bia B, Akoulitchev A (1996). A novel mutation in the promotor region in a family with a mild form of retinoblastoma indicates the location of a new regulatory domain for the *RB1 *gene. Oncogene.

[B31] Otterson GA, Modi S, Nguyen K, Coxon AB, Kaye FJ (1999). Temperature-sensitive RB mutations linked to incomplete penetrance of familial retinoblastoma in 12 families. Am J Hum Genet.

[B32] Dryja TP, Rapaport J, McGee TL, Nork TM, Schwartz TL (1993). Molecular etiology of low-penetrance retinoblastoma in two pedigrees. Am J Hum Genet.

[B33] Bremner R, Du DC, Connolly-Wilson MJ, Bridge P, Ahmad KF, Mostachfi H, Rushlow D, Dunn JM, Gallie BL (1997). Deletion of RB exons 24 and 25 causes low-penetrance retinoblastoma. Am J Hum Genet.

[B34] Schubert EL, Strong LC (1997). A splicing mutation in *RB1 *in low penetrance retinoblastoma. Hum Genet.

[B35] Lefevre SH, Chauveinc L, Stoppa-Lyonnet D, Michon J, Lumbroso L, Berthet P, Frappaz D, Dutrillaux B, Chevillard S, Malfoy B (2002). A T to C mutation in the polypyrimidine tract of the exon 9 splicing site of the *RB1 *gene responsible for low penetrance hereditary retinoblastoma. J Med Genet.

[B36] Weir-Thompson E, Condie A, Leonard RC, Prosser J (1991). A familial *RB1 *mutation detected by the HOT technique is homozygous in a second primary neoplasm. Oncogene.

[B37] Shapiro MB, Senepathy P (1987). RNA splice junctions of different classes of eukaryotes: sequence statistics and functional implications in gene expression. Nucl Acid Res.

[B38] Klutz M, Brockmann D, Lohmann DR (2002). A parent-of-origin effect in two families with retinoblastoma is associated with a distinct splice mutation in the *RB1 *gene. Am J Hum Genet.

[B39] Onadim Z, Hogg A, Baird PN, Cowell JK (1992). Oncogenic point mutations in exon 20 of the *RB1 *gene in families showing incomplete penetrance and mild expression of the retinoblastoma phenotype. Proc Natl Acad Sci U S A.

[B40] Dunnen JT, Antonarakis SE (2000). Mutation nomenclature estensions and suggestions to describe complex mutations: a discussion. Hum Mutat.

[B41] MySQL. http://www.mysql.com/.

[B42] PHP. http://www.php.net/.

[B43] JavaScript Kit. http://javascriptkit.com/.

[B44] FPDF Library. http://www.fpdf.org/.

[B45] Retinoblastoma gene mutation database (RBGMdb). http://www.es.embnet.org/Services/MolBio/rbgmdb/idx.html.

[B46] UK Human Genome Mapping Project. http://www.hgmp.mrc.ac.uk/.

[B47] Watakabe A, Tanaka K, Shimura Y (1993). The role of exon sequences in splice site selection. Genes Dev.

