# Supplementary material for: RB1 gene mutation up-date, a meta-analysis based on 932 reported mutations available in a searchable database
Source: BMC Genet. 2005 Nov 4;6:53. doi: 10.1186/1471-2156-6-53 (PMC1298292; doi:10.1186/1471-2156-6-53)
Supplement: Additional File 5 — Possible alternative splicing in the low-penetrance mutation c.607+1G>T [file 1471-2156-6-53-S5.doc]

**Additional file 5**

*Cis*-acting sequence elements involved in splicing of RB1 exon 6 and a cryptic exon 6.1 in mutation c.607+1G>T

|  | Branchpoint^a^ (CV) | Acceptor^a^  (CV) | Donor^a^  (CV) | ERS^b^ | Consequences |
| --- | --- | --- | --- | --- | --- |
| Exon 6  wt | gtgat (-45nt)  (68.4) | tatttgtttaatagG (83.0) | AAGgtaagt  (97.0) | 5´**GA**T**A**TCT**A**CT**GAAA**T**AAA**TTCT (11/11=1) |  |
| Exon 6  IVS6+1G>T | Same as wt | Same as wt | AAG**t**taagt  (58.1) | Same as wt | Skiping exon 6,  frameshift |
| Exon 6.1^e^  (NIX predicted) | ctgaa (-35nt)  (90.3) | aggatggttaccagA  (65.7) | GAGgtgagg  (80.5) | 5**ÁGG**CT**GGGAAGGG**T**GG**T**GGAGG** (18/4=4.5) | Inframe alternative splicing |

^a^Branchpoint, acceptor and donor splicing sites consensus values (CV) were obtained according to Shapiro and Senepathy [37]. The branchpoint sequence also gives the distance in nucleotides from the exon in brackets. .

^b^Exon recognition sequence (ERS) according to Watakabe et al., 1993 [47]with polypurine stretches underlined, and the ratio purine/pyrimidines in brackets.

^c^Cryptic exon 6.1 (g.52761-g.57293) predicted from computer analysis [46] of RB1 sequences comprising exons 5 to 7.

Skipping of exon 6 results in a premature termination and inactive Rb protein. However, in the mutated allele, the stronger branchpoint, donor splice site and exon recognition sequences will favour the usage of cryptic exon 6.1 which can be correctly spliced in frame:

exon 5 TC- A GGC TGG GAA GGG TGG TGG AGG TTT GGG AGA G-GC exon 7

The traslation product will be a 10 AA sequence as compared to 22AA in the normal sequence

Normal sequence: S ISTEIDSALVLKVSWITFLLAK G

Alternative sequence: S GWEGWWRFGR G (exon 6.1)

IVS6+1G>T

5

6

6.1 6.6.1

7
