# Supplementary material for: RB1 gene mutation up-date, a meta-analysis based on 932 reported mutations available in a searchable database
Source: BMC Genet. 2005 Nov 4;6:53. doi: 10.1186/1471-2156-6-53 (PMC1298292; doi:10.1186/1471-2156-6-53)
Supplement: Additional File 6 — Description of the flat-file format of RBGMdb. [file 1471-2156-6-53-S6.doc]

**Additional File 6:** Description of columns in the flat-file format

**(1) Location**: Exon (E) and intron (I) number according to cDNA sequence (NCBI, NM_000321.1)

**(2) Genomic:** Description follows the recommendations published by Dunnen and Antonarakis [40] using the genomic sequence GenBank: L11910.1

**(3) cDNA** changes as in Dunnen and Antonarakis [40], using the cDNA sequence NCBI: NM_000321.1.

**(4) Protein**: Deduced changes at the protein level follow the recommendations by Dunnen and Antonarakis [40] using the protein sequence NCBI: NP_000312.1.

**(5) Consequences**: predicted consequences are as follows: regulation (promoter), FS (truncating frame shift), IF (non-truncating in frame changes), MS (missense changes), NS (non-sense truncating mutations), SP (truncating mutations affecting splicing sites), SP-IF (in frame exon deletion due to splicing mutations), SP-MS (mutations affecting the last two nucleotides in exon can either be considered as MS or splicing mutations.

**(6) Type of mutation**: DUP (duplication) IN (insertion), DE (deletion), I_D (complex insertion and deletion), and PM (point mutation).

**(7) Origin**: Germ line or somatic.

**(8) Sample**: PB (peripheral blood) or FB (fibroblasts) for germ line, retino (retinoblastoma) and other (other tumours) for somatic mutations.

**(9) Phenotype**: B (sporadic bilateral), BF (bilateral familiar), U (sporadic unilateral), UF (unilateral familiar), UMF (unilateral multifocal), LP (familiar with low penetrance).

**(10) Sex**: F (female), M (male)

**(11) Age** at diagnosis or treatment in months.

**(12) Country** of origin of probands or of the main research group in publications, when the origin of probands was not reported

**(13) References** linked to PubMed abstracts

**(14) Patient_ID**, when available, as reported in publications.

**(15) Remarks**: any observation which can be useful in the context of a given mutation
